# Correction

**DOI:** 10.1093/geroni/igae032

**Published:** 2024-03-30

In the GSA 2023 Abstract Book as a supplement of *Innovation in Aging*, errors were noted in the following abstracts and have since been corrected:

Abstract Citation ID: igad104.1771: The abstract had a typo in the title (a missing space between “DEMENTIA” and “IN”). The correct title should be, “THE IMPACT OF THE COVID-19 PANDEMIC ON INFORMAL CAREGIVERS OF PERSONS WITH DEMENTIA IN THE POST-VACCINATION ERA.”

Abstract Citation ID: igad104.2432: The abstract had the incorrect title (duplicated title) of “ASSESSING SYMPTOM NETWORKS OF DEPRESSIVE SYMPTOMS IN OLDER ADULTS: A CROSS-NATIONAL POPULATION-BASED STUDY.” The correct title should be, “CROSS-SECTIONAL AND LONGITUDINAL ASSOCIATIONS BETWEEN SLEEP DISTURBANCES, DEPRESSIVE AND ANXIETY SYMPTOMS.”

Abstract Citation ID: igad104.2578: The abstract had a typo in the title (an extra “T” instead of a space; “ANDTTHEIR”). The correct title should be: “CHILDHOOD TRAUMA AMONG US VETERANS AND THEIR NEGATIVE HEALTH AND RISKY BEHAVIORS IN LATER LIFE.”

https://doi.org/10.1093/geroni/igad104.1771

https://doi.org/10.1093/geroni/igad104.2432

https://doi.org/10.1093/geroni/igad104.2578

